# Concerns and educational needs of Iranian parents regarding the sexual health of their male adolescents: a qualitative study

**DOI:** 10.1186/s12978-020-0883-6

**Published:** 2020-02-14

**Authors:** Shahnaz Babayanzad Ahari, Zahra Behboodi Moghadam, Seyed Ali Azin, Raziyeh Maasoumi

**Affiliations:** 1 Reproductive Health Department, School of Nursing and Midwifery, Tehran University of Medical Science, Tehran, Iran; 2 Reproductive Biotechnology Research Center, Avicenna Research Institute, ACECR, Tehran, Iran

**Keywords:** Sexuality education, Adolescent, Parents’ concerns, Qualitative study, Iran

## Abstract

**Background:**

Parents play an important role in promoting the sexual health of their adolescents. However, many parents experience several challenges. The purpose of this study was to explore the concerns and educational needs of Iranian parents regarding the sexual health of their male adolescents.

**Methods:**

This qualitative study was designed based on the conventional content analysis approach. Semi-structured and in-depth interviews were conducted with 16 parents of male adolescents aged 12–18 years. All interviews were audio recorded and transcribed verbatim. The data were collected through purposeful sampling and continued until data saturation. Finally, the Graneheim and Landman strategies were used to analyze data.

**Results:**

According to the participants’ comments, four main categories were extracted as follows: fear of emotional and sexual harms, quality of parent-child relationships, effect of media and cyberspace, and necessity of sexuality health education.

**Conclusions:**

The findings highlighted the need for sexuality health education through cooperation with schools for offering appropriate education to the students, parents, and school staffs.

The results showed that parents required training to enhance their knowledge and skills to improve their communication with their adolescents about sexuality issues. Therefore, it is necessary to design, implement, and evaluate culture-appropriate educational programs to address the parents’ concerns regarding adolescents’ sexual health.

## Plain English summary

Parents play a key role in shaping the sexual behavior of adolescents. They are a primary source of values, morals, and standards which have a great influence on children’s sexual socialization and decision making. This qualitative study aimed to explore concerns and educational needs of parents regarding the sexual health of their male adolescents in the Iranian cultural context. The findings of this study will provide ideas for design and implementation of educational programs to address parents’ concerns regarding sexual health of male adolescents. Semi-structured and in-depth interviews were conducted with 16 parents of male adolescents aged 12–18 years between February 2018 and August 2018 in Karaj, Iran. According to the participants’ comments, four main categories were extracted as follows: fear of emotional and sexual harms, quality of parent-child relationships, effect of media and cyberspace, and necessity of sexuality health education. The participants highlighted that they need more support, knowledge, skills and resources to manage adolescents’ sexuality. In another study, we are going to design and implement an educational intervention for Iranian parents.

## Background

Adolescence is a phase of rapid biological and psychosocial maturity changes that have health effects throughout the lifelong [1]. The importance of adolescent sexual health has been highlighted well in the last two decades [2]. According to available data from most countries, sexual activities begin in the early adolescence [3]. Engaging adolescents in sexual behavior without appropriate knowledge and skills can increase the risks of poor sexual health outcomes, including unwanted pregnancy, HIV, and sexually transmitted infections [4]. The World Health Organization (WHO) emphasizes that sexuality education is the best way for adolescent to learn safe sexual behaviors and avoid risky sexual behaviors [5]. The goals of comprehensive sexuality education include not only prevention, but also wellbeing and healthy decision making of young people [6].

Parents play a key role in shaping the sexual behaviors of young people. They are a primary source of values, morals, and standards which have a great influence on children’s sexual socialization and decision making [7]. Some research studies indicated that the effective parents-adolescent communication about sexual issues has been associated with delaying onset of sexual activity, greater contraceptive use, and fewer sexual risk behaviors [8–10].

It is noteworthy that fulfillment of the parenting duty is not easy. Parents might experience several challenges about when, what, and how to talk to their children about puberty, reproduction, and sexuality [11]. Many parents do not acknowledge their adolescents as sexual beings or may underestimate their engagement in sexual activity so they may not see the need to discuss [12, 13]. In a review study has been conducted with American or European samples, barriers to parental communication about sex have been attributed to demographic factors (like cultural, political, and religious factors), inadequate knowledge, embarrassment or feeling uncomfortable when talking about sexuality, and a belief that their adolescent was not ready to talk about sexuality [14]. Among parents who wanted to provide sexuality health education to their children, many preferred to focus on concerns related to cautions and negative consequences of sexuality (e.g., STIs, risks of pregnancy, and sexual assault) rather than positive and developmental aspects (pleasure and romantic relationships) [15, 16]. Some other researchers identified that majority of parents do not begin discussion about sex until their adolescent is engaged in a relationship or when a negative consequence is disclosed [17, 18]. Globalization and developments in recent years have emerged a new need of sexuality education for young generation as concerns accessing to so many sources of sexualized information, changing patterns of sexual behaviors, sexual rights, and the threat of HIV and other STIs [4].

Iran, along with other countries, witnesses considerable changes in the socio-cultural context in which young people are making decision for their own sexual behaviors [19]. Sexual relationships before marriage are not accepted due to cultural and religious aspects in Iran. However, because of gender-based double standards among male and female adolescents in Iranian families, male adolescents have reported earlier premarital sex and more sexual partners than females, which had often been unsafe [20]. In this regard, the result of a study in Iran has indicated that 28% of male adolescent aged 15–18 years have had sexual experience before the marriage [21]. Moreover, Zare et al. (2017) reported that sexual health had been the main health needs of adolescent boys in Iran [22]. Despite the essential need of sexual health education for Iranian adolescents, there are no school-run programs in their educational curriculum yet [23]; thereby many of adolescents do not obtain proper sexual education in schools. Now pressure is upon parents, as it is their responsibility to have more active role in sexual health education. According to a recent study conducted in Iran, mothers of female adolescents believed that they could be better sexual health trainers for their girls [24]. In respect of this issue, parents’ concerns and needs should be addressed seriously by specialists. Because of the contextual base of sexuality, this qualitative study was the first study aimed to explore concerns and educational needs of parents regarding the sexual health of their male adolescents in an Iranian cultural context. The results of this study will provide ideas for design and implementation of sexuality education programs for Iranian parents.

## Methods

### Study design and participants

The current study was the preliminary part of a study with multistage mixed methods design which highlighted concerns and educational needs of parents regarding the sexual health of their male adolescents in an Iranian context for the first time. This study was a qualitative study that was conducted through a conventional content analysis approach. The participants were parents of male adolescents in 7th–12th grade (about ages 12–18 years) from different all-boys high schools with Iranian nationality in Karaj city. Karaj is a metropolitan city with a population around 1.97 million. It was ranked as the fourth most populous city in Iran according to the Statistical Center of Iran reports in 2016 [25]. The participants consisted of 16 parents (9 mothers and 7 fathers) that were recruited based on purposeful sampling. Different perceptions from parents were achieved with maximum variation strategy in terms of gender, age, education, occupation, marital status, economic status, number of children, adolescent’s age, and residency region.

### Data collection

The data were collected between February 2018 and August 2018. Semi-structured and in-depth interviews were used in this study. Interview is an effective collection technique for obtaining in-depth information from participants on their experiences with their own words in an open way [26]. Interviews were conducted in schools or the parents’ work place for their convenience. The interviewer was a Ph.D. candidate in Sexual and Reproductive Health. All interviews were individual, face to face and done in a private room. The average time of each interview was about 60 min and all were audio recorded with participants’ permission. The interviews ended when data saturation was reached. MAXQDA-10 software (VERBI GmbH, Marburg, Germany) was used for data management. All the interviews started with general questions then followed by probing questions to achieve more detailed information. Some of the questions were as follows:

- Could you tell me your thoughts about the sexual health of your son?
- Would you tell me how you feel about the sexual health of your son?
- What is your idea about sexual health education for adolescents?
- How do you imagine your role in providing sexual education for your son?
- What would help you in order to provide sexual education information to your son?
- Further probing questions were asked to clarify explanations, including: Could you give me an example? / may you explain more?

### Data analysis

The data were analyzed based on Graneheim and Landman strategies (2004). The transcripts were read several times to get a general concept. The meaning units were identified and then the related ones were condensed and converted to codes. Eventually, codes were classified into categories and subcategories [27].

### Trustworthiness

Credibility, dependability, conformability, transferability, and authenticity were considered for the trustworthiness of the results [28]. Credibility of the study results was strengthened through spending enough time on data collection, diversity of participants, and member checking, in which, the transcripts and codes were returned to participants for clarifying any ambiguous codes. Dependability and conformability was enhanced through external check and peer debriefing; therefore, the research team including two expert qualitative researchers and two external supervisors reviewed and rechecked the transcripts, codes, and categories in order to find any disagreement in the coding process. We carried out purposeful sampling with maximum variation for transferability. Authenticity was established by member checking and participants compared the results of the research team with their own views. Eventually, two different persons translated the interviews from Persian to English and a third person confirmed the translated version of texts.

## Results

Sixteen parents (9 women and 7 men) participated in this study. The participants ranged from 33 to 52 years old with a mean age of 43.18 (±4.9) years. The demographic information of participants is presented in Table 1. Four main categories with 10 subcategories have been identified. The main categories included: fear of emotional and sexual harms, quality of parent-child relationships, effect of media or cyberspace, and necessity of sexual health education (Table 2).

**Table 1:** Demographic Characteristics of Participants (*N* = 16)

Characteristic	Participants
Age (mean ± SD)	43.18 ± 4.9
Gender(N. %)	
Male	7(44)
Female	9(56)
Education level (N. %)	
High School	2(13)
Diploma	5(31)
University	9(56)
Occupation (N. %)	
Employed	11(69)
Unmployed	5(31)
Family status (N. %)	
Couple parent	12(75)
Single parent (divorce/death)	4 (25)
Socio-Economic Status (N. %)	
Good	6(37)
Average	7(44)
Weak	3(19)
Number of male adolescents(N. %)	
1	13(81)
2	3(19)
Adolescent’s age (N. %)	
12–14	7(44)
15–18	9(56)
Adolescent’s grade (N. %)	
Primary High School (7th–9th)	9(56)
Secondary High School (10th-12 th)	7(44)

**Table 2:** Categories and Sub-categories

Categories	Sub-categories
Fear of emotional and sexual harms	- Parents’ fear of sexual harassment in adolescents - Parents’ concerns about adolescents’ emotional and sexual behaviors
Quality of parent-child relationships	- Weak interpersonal relationships in family - Inability to communicate with children about sexuality issues
Effect of media and cyberspace	- More exposure to sources of sexual stimuli - Lagging behind the technology
Necessity of sexuality health education	- Sexuality health education for students - Sexuality health education for teachers, instructors, and school authorities - Sexuality health education for parents - Participation of family and school in sexuality health education

### Fear of emotional and sexual harms

The results showed that one of the parents’ concerns was vulnerability of adolescents to consequences of emotional and sexual relationships. This category was composed of two subcategories as follows:

#### Parents’ fear of sexual harassment in adolescents

This issue was one of the major concerns of parents expressed by most of the participants. Such threats are now a growing concern among Iranian families. One of the parents, who had a 12-year-old son, said:

“*Incidents like sexual assault are my serious concern. I want the society to be safe, so that nothing would happen to my children that are irreparable or hard to repair*” (p.1, 45 y).

A mother of a 15-year-old boy expressed her concerns about adolescents’ lack of proper perception of dangers, and stated:

“*… I fear that they might be abused. They are confused at this age and may not understand they are being abused”* (p.5, 42 y).

#### Parents’ concerns about adolescents’ emotional and sexual behaviors

While on the one hand parents were worried about the consequences of sexual and romantic relationships of their adolescents; on the other hand, they were concerned about the slow or abnormal growth of their children’s sexual orientation and behavior. One of the mothers mentioned about her concerns regarding sexual relationship of her 18-year-old son and said:

“*… He may have a girlfriend and do something that is embarrassing. AIDS is prevalent and it is not clear with which girl he is involving …*” (p.4, 43 y).

A mother of a 15-year-old boy also stated:

“*… Since my son is the only of mine, I do not know the appropriate reaction if he is involved in romantic relationships. I do not know what I should do if something happens or how to prevent it from happening and how to put it in the right way if it happens”* (p.6, 43 y).

One of the fathers who had a 13-year-old- son said:

“*… I am worried about my son that may have sexual abnormalities; for example, he may incline to the same sex or has no sexual desire or has extraordinary sexual desire”* (p.3, 45 y).

However, some of the fathers had more permissive attitude towards boys. A father of 12-year-old boy explained in this regard:

“*… It is important to keep these feelings at a normal level. Personally, I give my child the opportunity and even I prepare the way for him to enjoy sexual pleasure at an ordinary level”* (p.1, 45 y).

### Quality of parent-child relationships

Most of the parents were dissatisfied with their lack of communication skills in general and about sexuality issues in particular. This category had two subcategories:

#### Weak interpersonal relationships in family

The major concerns of the participants in this subcategory were difficulty in establishing a friendly communication with their children, lack of control over children’s activities*,* family structure instability*,* and replacement of real communications with virtual relationships.

A mother that had two boys aged 16 and 19 years, gave her opinion saying:

“*… When you are friend with your children they also feel relaxed and avoid many bad things. I have seen the parents who are on friendly terms with their children, are more successful in many aspects”* (p.7, 42 y).

A young mother who had a second marriage and her 13-year-old son did not live with her, stated: “*… I see my son on weekends. I would be less worried if my son lived with me because I think I could control him better …*” (p.10, 33 y).

A mother of 12-year- old boy complained about the impact of increased virtual relationships on family connections and said:

*“You see now many of children have access to the computer, laptop, and smart phone easily which weaken their relationship with family members …*” (p.9, 46 y).

#### Inability to communicate with children about sexuality issues

Many of the parents had challenges about effective communication with their adolescents regarding sexual matters that mostly resulted from their lack of knowledge, inadequate skills and comfort in talking about sexual matters, and their beliefs or attitudes about sexuality.

One of the mothers who were a teacher in boys’ school was complaining of her lack of knowledge due to not receiving sexuality education, explained:

*“We were a generation that never discussed these things with our parents. It was very obscene. We had no information, even in our schoolbooks. When I discovered my son was going through puberty, I as a mother felt I needed to talk with a counselor”* (p.2, 40 y).

A father in the position of boys’ school principal, also mentioned to his inability in establishing an effective communication with his 18-year-old son, and said:

“*… My son is now a grown-up but we have not been able to talk about these things even for 30 seconds. They may have sexual problems like all other problems. They should feel comfortable enough to turn to their parents to discuss it”* (p.15, 50 y).

Another father who had an 18-year-old son expressed his opinion about cultural beliefs in these words:

“*… In our culture, parents think that their children will be impudent if they speak with them frankly and they may no longer listen to their parents”* (p.8, 47 y).

### Effect of media and cyberspace

Most of the parents in our study believed that the world of their children was thoroughly different from the world they grew up. They had many concerns about their teenagers’ exposure to the sexual content of the social media and new technologies and their effects on their attitude and behavior. On the other hand, they believed that they had little control over this environment due to their little knowledge compared to their children’s information. This category had two subcategories:

#### More exposure to sources of sexual stimuli

Many of the parents believed that their children’s exposure to sexually explicit content of movies, pictures, and information through the satellite, mobile phones, and the Internet. They were afraid social media could affect their sexual behavior. Some participants were of the opinion that the teenagers were more exposed to these conditions in some families. A mother of two boys aged 14 and 17 years, said:

“*… Well, unfortunately, with this internet and the available web pages, there is no framework for the information available to children. They may search a name and receive a great deal of information”* (p.12, 36 y).

A mother of 12-year-old boy asserted:

*“The parents who leave their teenagers at home alone that there is high speed internet and satellite receiver in access and the teenager is going through puberty, many things may happen that shouldn’t …*” (p.14, 46 y).

#### Lagging behind the technology

The parents believed in an increased gap between the two generations and information bombardment of the teenagers and emphasized the need for updating their technological information along with their children. A mother of 15-year-old boy explained her experience as follows:

“*… I think we are way behind the world that our children are living in. We have little information and our children are far ahead of us”* (p.9, 45 y).

### Necessity of sexuality health education

Most of our participants believed that sexuality health education is a necessity in today’s society and placed a great deal of emphasis on the role of family and school in this regard. This category had four subcategories.

#### Sexuality health education for students

Many of the parents believed that school should play an active role in teaching sexuality health to teenagers. They discussed the necessity of involving schools in sexuality health education, absence of sexuality health education in schools, and educations being limited to scientific lessons. A participant who was a father of 18-year-old boy said*:*

“*… Even if the children do not receive the necessary training from the family, they should be guided in the right path by the school. If the school forbids, the teacher forbids, then who should give information to our children?”* (p.16, 44 y).

Another mother of 13-year-old boy stated:

“*… Our schools are unfortunately weak in this regard. I think it is much more important than mathematics and dictation...”* (p.6, 43 y).

#### Sexuality health education for teachers, instructors, and school authorities

Some of the parents complained the lack of sexuality health education for teachers, instructors, and school authorities. A mother of 12-year-old boy mentioned in this regard:

“*… This education is necessary and should start from teachers to learn the limits of what they should say, and that they should not answer every question”* (p.14, 46 y).

#### Sexuality health education for parents

Majority of the parents stated that they required knowledge and skills to manage the adolescents’ sexuality issues properly but they did not have access to valid sources. A mother of 18-year-old boy emphasized the need for sexuality health training courses for parents and proposed: “*… It is very important that parents receive training. I think this training should be practical, like holding classes, running workshops, and etc”* (p.4, 43 y).

A father of 13-year-old pointed to the scarcity of valid sources about sexuality health education and declared:

*“There should be valid sources for information. Mass media do not give the necessary information. Where should I acquire this knowledge? There are no classes, no media. I have to search on the internet but my efforts have been in vain.” (*p.11, 37 y).

#### Participation of family and school in sexuality health education

Some of the parents underlined the importance of the interaction of teachers and counselors with teenagers and families in sexuality health education. A mother of 15-year-old boy who was a Nurse, in this regard said:

“*… I wish there was a stronger relationship (between school and parents). Teachers should say that we discussed these issues with your children and you follow this path at home. There is unfortunately no link between parents and teachers in such matters”* (p.5, 42 y).

## Discussion

In current study, sexual harassment of adolescents had been a major concern of the parents; especially those who had boys in early adolescence. Covering the news of sexual assault in the media had made the participants more sensitive to this issue. This was also evident in Australian parents who expressed their concern about the potential of children exploitation or sexual abuse [11]. According to the World Health Organization (WHO) report, about 20% of the females and 8% of the males have experienced the sexual abuse before the age of 18 [29]. There are some evidences that parental involvements in young people’s private lives are significant factor for prevention of sexual assaults [30]. Since parents in our study have realized the importance of this issue, it could be an entry point for parents’ participation in sexuality education.

Emotional and sexual behaviors of adolescents were another challenge of parents in our study. Parents whose children were at the beginning of adolescence were more worried about abnormal sexual development, gender identity, and homosexual desire while the parents whose children were at the end of adolescence were concerned about the consequences of being involved in romantic and sexual relationship with the opposite sex. Parents’ attitude towards sexual behaviors has a significant effect on the adolescents’ decision making about their sexual activity [7]. The point worth mentioning here are the differences between parents in regard to the concept of sexuality in various contexts [31]. Some parents believe in abstinence and impose more control over their adolescents’ sexual behavior; or some may look sexuality as a normal activity and permit adolescents more autonomy to make decisions on their sexuality [31, 32]. Obviously, there are more risks for adolescents who engage in sexual behaviors without proper knowledge and skills [4]. Thus, it is necessary that parents receive information about normal development of adolescent sexuality for proper managing of these behaviors and protecting them from sexual risks.

The quality of the parent-child relationships was another category which the most of parents in our study reported certain challenges. They highlighted weak interpersonal relationships due to some difficulties in establishing a friendly communication with their children and/or control over their children’s activities. According to studies, the ways that parents interact with their children, as well as the strategies that they develop to protect them from specific risks have considerable influence on children’s sexual behavior [32]. Dittus et al. (2015) in a meta-analysis study reported that, parental control over adolescents was associated with delayed onset of sexual relationship or use of contraceptive methods [33]. Inability to communicate and talking about sexuality issues with adolescents was another important point according to the findings of this study. The parents believed that it was due to lack of knowledge, inadequate skills and comfort in talking about sexual matters, and their beliefs or attitudes about sexuality. The pubertal transition often disturbs the relationship patterns so that discussions about sexual issues may be difficult [34]. Afifi et al.’ study (2008) in the United States indicated that parents’ lack of communication skills contributed to fear of sexual discussions in adolescents and greater avoidance as a result [35]. In line with the studies by Ballard and Gross (2009) in the United States and Hyde et al. (2013) in Ireland, our research confirmed that most of the parents were not satisfied of not receiving sexual education during their own adolescence and implied that they intended to break their silence and establish a more effective and comfortable dialogue with their children about sexuality through acquiring the necessary knowledge and skills [17, 36]. Similarly, another study conducted in Iran showed that 63% of the parents agreed with talking about sexuality with their adolescent males [21]. Thus, along with adolescents’ developmental changing, parents’ approach to discussing sex with them should be changed [34]. In this regard, improving parent-adolescent general relationship, eliminating the barriers to effective communication, and promoting sexual communication via appropriate content and quality are necessary [14].

The participants in our study believed that today’s world was so different from the world in which they had grown up. They stated that teenagers were technologically smart and the parents could never catch up with them in the information age; on the other hand, they had major concerns about their teenagers’ exposure to the sexual content of the social media and new technologies. The existing evidence concerning the effects of pornography on adolescents’ sexual behavior is characterized by mixed findings. A survey in the United States in 2009 indicated that 85% of adolescent males and 50% of adolescent females had been exposed to pornography and sexual experiences were more common among them [37]. In another study conducted in Iran, was shown that there was a relationship between pornography and sexual behaviors in adolescents, especially boys [38]. In contrast, the results of two studies in Swiss and Croatia contexts showed that the majority of the high-risk sexual behaviors in adolescents had no relationship with exposure to pornography [39, 40]. In this regard Bailin et al. (2014) argued that adolescent education has a more important role and parents need training to be updated against the effects of social networks [41].

Most participants of this study believed that sexuality health education was a necessity in the today’s society and have positive view about it. The parents, especially those with lower economic or education level, were willing that school should be pioneer of sexuality health education because they believed that schools could provide young people with more reliable information. In Iran as a conservative country, there are still some cultural barriers to sexuality health education promotion for the adolescents [23]. Despite the opposition of some political and religious groups with sexuality education, parents in many parts of the world support sexuality health education in schools [42, 43]. However, some parents may have concerns due to limited knowledge, nature of sexuality education, and society norms [6]. A number of participants in our study pointed to educational programs for teachers and school staff so that they can deliver appropriate and safe sexual information and training for adolescents. This result was consistent with the study of Dyson et al. (2010) which parents wanted to be assertive that their children’s educators had enough skills and qualifications [11]. Most of parents in our study emphasized their own role, but they highlighted that they require more resources and training materials. In a survey conducted in Australia, one-third of the parents sought more sexuality education resources for preparation [44]. Furthermore, our parents believed that sexuality education is a mutual process between the home and the school, requiring close cooperation of teachers, counselors, and parents; so they proposed that in every school, a counselor should be in contact with adolescents and parents on issues relating to sexuality. Involvement of parents in school sexuality education may be an important point to ensure that young people receive appropriate information [6]. The results of a study on Tanzanian parents revealed that adolescents’ sexuality education is more effective when parents’ perspectives and values and are considered in the program [43]. However, more research is needed to determine the school-family partnerships [11]. Overall, the qualitative design of this study provided an opportunity to discover insights and feelings of Iranian parents. As mentioned earlier, parents do not feel empowered regarding adolescents’ sexuality and need more support, knowledge, skills, and resources to deal with it. In a follow up study, we intend to design and implement an educational intervention for Iranian parents. A potential limitation of study was a small number of participants in a single city; therefore, the results could not necessarily be generalized to other Iranian parents.

## Conclusions

The results of this study revealed that today’s world is more challenging to Iranian parents due to the greater exposure of adolescents to sexual stimuli. The participants highlighted to keep up with development in the society more educational approaches are needed to improve adolescents’ sexual health. This can be achieved through cooperation with schools for offering appropriate and relevant education to the students, parents, and school staff. Meanwhile, it is also necessary to call for the cooperation of policymakers, ministry of education, ministry of health, media, and religious missionaries. The results highlighted that the parents are dissatisfied with their lack of information and communication skills in sex-related issues, as well as limited access to training resources. Therefore, the results provide the evidence to design, implement, and evaluate culture-appropriate educational programs to address parents’ concerns regarding sexual health of their male adolescents.

## Data Availability

The data set are available from the corresponding author on reasonable request.
